# Arrhythmic Risk Stratification by Cardiovascular Magnetic Resonance Imaging in Patients With Nonischemic Cardiomyopathy

**DOI:** 10.1016/j.jacc.2024.06.046

**Published:** 2024-10-08

**Authors:** Daniel J. Hammersley, Abbasin Zegard, Emmanuel Androulakis, Richard E. Jones, Osita Okafor, Suzan Hatipoglu, Lukas Mach, Amrit S. Lota, Zohya Khalique, Antonio de Marvao, Ankur Gulati, Resham Baruah, Kaushik Guha, James S. Ware, Upasana Tayal, Dudley J. Pennell, Brian P. Halliday, Tian Qiu, Sanjay K. Prasad, Francisco Leyva

**Affiliations:** a National Heart and Lung Institute, Imperial College London, London, United Kingdom; b Royal Brompton & Harefield Clinical Group, part of Guy’s and St Thomas’ NHS Foundation Trust, London, United Kingdom; c Kings College Hospital NHS Foundation Trust, London, United Kingdom; d University Hospitals Birmingham Queen Elizabeth, Birmingham, United Kingdom; e Aston Medical School, Aston University, Birmingham, United Kingdom; f Anglia Ruskin Medical School, Chelmsford, United Kingdom; g Essex Cardiothoracic Centre, Basildon, Essex, United Kingdom; h MRC Laboratory of Medical Sciences, Imperial College London, London, United Kingdom; i Department of Women and Children’s Health, King’s College London, London, United Kingdom; j British Heart Foundation Centre of Research Excellence, School of Cardiovascular and Metabolic Medicine and Sciences, King’s College London, London, United Kingdom; k Lewisham and Greenwich NHS Trust, London, United Kingdom; l Portsmouth Hospitals NHS Trust, Portsmouth, United Kingdom

**Keywords:** arrythmia, fibrosis, nonischemic cardiomyopathy, risk stratification, sudden cardiac death

## Abstract

**Background:**

Myocardial fibrosis (MF) forms part of the arrhythmic substrate for ventricular arrhythmias (VAs).

**Objectives:**

This study sought to determine whether total myocardial fibrosis (TF) and gray zone fibrosis (GZF), assessed using cardiovascular magnetic resonance, are better than left ventricular ejection fraction (LVEF) in predicting ventricular arrhythmias in patients with nonischemic cardiomyopathy (NICM).

**Methods:**

Patients with NICM in a derivation cohort (n = 866) and a validation cohort (n = 848) underwent quantification of TF and GZF. The primary composite endpoint was sudden cardiac death or VAs (ventricular fibrillation or ventricular tachycardia).

**Results:**

The primary endpoint was met by 52 of 866 (6.0%) patients in the derivation cohort (median follow-up: 7.5 years; Q1-Q3: 5.2-9.3 years). In competing-risks analyses, MF on visual assessment (MF_VA_) predicted the primary endpoint (HR: 5.83; 95% CI: 3.15-10.8). Quantified MF measures permitted categorization into 3 risk groups: a TF of >0 g and ≤10 g was associated with an intermediate risk (HR: 4.03; 95% CI: 1.99-8.16), and a TF of >10 g was associated with the highest risk (HR: 9.17; 95% CI: 4.64-18.1) compared to patients with no MF_VA_ (lowest risk). Similar trends were observed in the validation cohort. Categorization into these 3 risk groups was achievable using TF or GZF in combination or in isolation. In contrast, LVEF of <35% was a poor predictor of the primary endpoint (validation cohort HR: 1.99; 95% CI: 0.99-4.01).

**Conclusions:**

MF_VA_ is a strong predictor of sudden cardiac death and VAs in NICM. TF and GZF mass added incremental value to MF_VA_. In contrast, LVEF was a poor discriminator of arrhythmic risk.

Nonischemic cardiomyopathy (NICM) is a common cause of heart failure. After presentation, the 5-year mortality approaches 38%.^1^ Although pump failure is the most frequent cause of death, sudden cardiac death (SCD) caused by ventricular arrhythmias (VAs) accounts for up to one-third of all deaths.^2^

As in ischemic cardiomyopathy (ICM), current guidelines recommend implantable cardioverter-defibrillators (ICDs) for the primary prevention of SCD in patients with NICM and a left ventricular ejection fraction (LVEF) of <35%.^3^, ^4^, ^5^ The use of LVEF in these guidelines stems from its adoption among the inclusion criteria in randomized controlled ICD trials. However, LVEF has never been shown to be a reliable predictor of VAs in either ICM or NICM.^6^ Accordingly, most patients with an LVEF of <35% who receive ICDs for primary prevention do not receive ICD shocks.^7^ In addition, most patients who succumb to SCD would not have fulfilled indications for ICD implantation.^8^, ^9^, ^10^ DANISH (Defibrillator Implantation in Patients With Nonischemic Systolic Heart Failure) showed no survival benefit from ICDs in patients with NICM who are selected according to LVEF.^11^ The limitations of LVEF as a predictor of VAs have been recognized by the National Heart, Lung, and Blood Institute; the Heart Rhythm Society^12^; and international clinical guideline groups.^4^^,^^5^

It is now well established that myocardial fibrosis (MF) forms part of the arrhythmic substrate for VAs.^13^, ^14^, ^15^ Numerous studies have shown that MF, assessed using cardiovascular magnetic resonance (CMR), is useful in arrhythmic risk stratification.^16^, ^17^, ^18^, ^19^, ^20^, ^21^, ^22^, ^23^ In this context, areas of maximal signal intensity on late gadolinium enhancement correspond to dense MF, whereas areas of intermediate signal intensity correspond to so-called gray zone fibrosis (GZF). In clinical outcome CMR studies, both total fibrosis (TF)^18^^,^^19^ and GZF^24^, ^25^, ^26^, ^27^ have emerged as risk factors for VAs. In this study, we explore whether TF and GZF predict SCD or VAs in patients with NICM across a wide range of LVEFs. We also explore whether quantification of these MF measures adds to visual assessment in arrhythmic risk stratification.

## Methods

### Study population

This is an observational study of patients with NICM from 2 large UK tertiary referral hospitals. The derivation cohort consisted of prospectively enrolled, consecutive patients from the Royal Brompton Hospital, London, United Kingdom. The validation cohort included retrospectively enrolled patients from University Hospitals Birmingham, Queen Elizabeth, Birmingham, United Kingdom. Some of the patients in the derivation cohort were included in previous publications,^28^^,^^29^ but the present study involves a longer follow-up and de novo quantification of TF and GZF. Recruitment to the prospective derivation cohort began in September 2009, and the first patient in the retrospective validation cohort was scanned in July 2010. The present study was conceived in 2022 after the senior investigators (F.L. and S.K.P.) agreed that the same data had been collected prospectively in the derivation cohort and retrospectively in the validation cohort. In light of the similarity of these cohorts, after agreement on the scope of the study and a strategy for data analysis, raw data from both centers were submitted to a statistician (T.Q.). Ethics Committee approval for the derivation cohort was obtained from the South Central Hampshire Research Ethics Committee (reference: 19/SC/0257). Approval from the Clinical Audit Department for the validation cohort was obtained from University Hospitals Birmingham (reference: CARMS 14153).

### Eligibility

Inclusion criteria for both cohorts were the following: dilated cardiomyopathy; hypokinetic, nondilated left ventricular (LV) cardiomyopathy; isolated LV dilatation; and/or late gadolinium enhancement consistent with NICM.^1^ Exclusion criteria were the following: history of ischemic heart disease or coronary revascularization, coronary angiography showing at least 1 >50% stenosis in a major epicardial coronary artery, inducible ischemia on functional testing, subendocardial or transmural pattern of late gadolinium enhancement consistent with a myocardial infarction, uncontrolled hypertension, primary valve disease, congenital heart disease, active myocarditis, active or quiescent cardiac sarcoidosis, infiltrative cardiomyopathy, channelopathies, and athletic remodeling. Genetic testing was not uniformly or widely applied during the study period, so we cannot quantify the proportion of patients with genetic cardiomyopathies (eg, titin, lamin a/c, and so on).

### Cardiovascular magnetic resonance

All patients underwent a CMR scan on a 1.5-T scanner (derivation cohort: Sonata and Avanto, Siemens; validation cohort: Magnetom Symphony and Avanto, Siemens). Long- and short-axis cine images were obtained using breath-hold, steady-state free precession sequences. Gadopentetate dimeglumine or gadobutrol (0.1 mmol/kg) was injected intravenously, and an inversion recovery gradient echo sequence was undertaken to acquire the late gadolinium enhancement images at 10 minutes. These images were acquired in long- and short-axis slices (8-mm slice thickness with a 2-mm gap) covering the LV from base to apex. Inversion times were optimized to null normal myocardium. Myocardial fibrosis on visual assessment (MF_VA_) was regarded as present when seen in both long- and short-axis images, in 2 orthogonal views, extending beyond the right ventricular insertion points, according to the reporting investigator and independently verified by a blinded observer.

**Figure 1:**
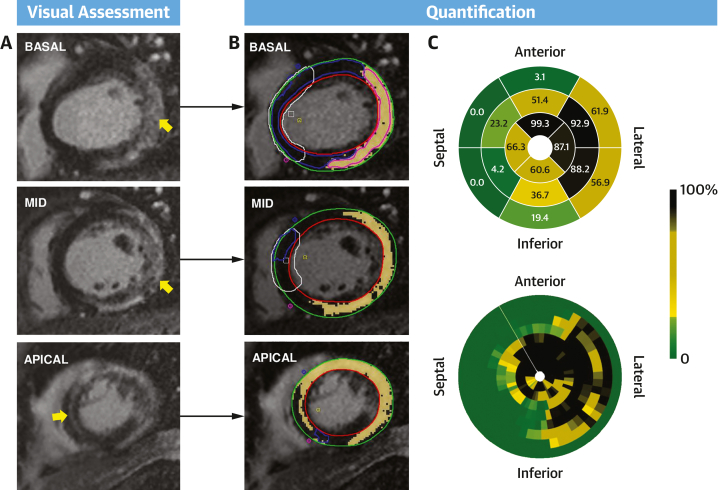
Cardiac Magnetic Resonance in Nonischemic Cardiomyopathy (A) Late gadolinium enhancement cardiovascular magnetic resonance images were visually assessed to determine whether myocardial fibrosis (MF) was present or absent. If MF was present (appears white on late gadolinium enhancement), quantification was undertaken. (B) To this end, epicardial and endocardial contours (green and red, respectively) were semiautomatically delineated. Total fibrosis and gray zone mass were quantified using various signal thresholding methods. In this example, the basal segments showed extensive, heterogeneous MF (yellow arrows) in a noncoronary distribution over the left ventricular free wall, with a distinct epicardial and midmyocardial distribution toward the midventricular and apical segments. (C) The polar maps show the distribution of MF according to the American Heart Association 16-segment model and to smaller segments (100 segments over 8 short-axis slices, starting from the junction of the right ventricular wall and the interventricular septum [white line]). The scale range is from 0 (green, no MF) to 100% (black, entire segment is 100% MF).

CVI42 software (Circle Cardiovascular Imaging Inc) was used to quantify TF and GZF mass. This was undertaken by 2 independent investigators (E.A. for the derivation cohort and A.Z. for validation cohort) who were blinded to clinical outcomes. Endocardial and epicardial contours were semiautomatically drawn on short-axis CMR images and manually optimized, excluding the blood pool and epicardial fat. Two regions of interest were defined using a semiautomated detection algorithm with manual adjustment: remote myocardium, defined as regions with no enhancement, and the region of hyperintense myocardium. TF mass was derived by signal threshold vs reference myocardium methods using the mean ± SD of the remote myocardial signal intensity at 2-SD, 3-SD, and 5-SD thresholds. TF mass was also calculated using the full-width half-maximum method (Figure 1). GZF mass was calculated as the difference between the MF mass using the 2-SD method and the 3-SD, 5-SD, and full-width half-maximum methods, termed GZF_3SD_, GZF_5SD_, and GZF_FWHM_, respectively, as previously described.^30^ In cases where no fibrosis was detected by visual assessment, all TF and GZF measures were imputed as 0. Total TF and GZF volumes were calculated by multiplying the enhanced area by slice thickness. Myocardial mass was calculated by multiplying volume in milliliters by the myocardial density (1.055 g/mL).

### Follow-up and endpoints

Patients were followed up using primary care and hospital records and from postal questionnaires sent to patients. Follow-up duration was measured from the date of CMR and truncated after 10 years. All clinical events were adjudicated by an independent panel of cardiologists for both cohorts using medical records; and, where available, death certificates, autopsy reports, coroners’ reports, and cardiac implantable device interrogation reports. Survival status was checked with the National Health Service Spine system, which links with the Office of National Statistics. Remote monitoring was not systematically used during the study period. All device interrogations were undertaken according to each center’s protocol. All potential arrhythmic events and cardiac device downloads were reviewed by an implantable cardiac devices expert (derivation cohort: K.G.; validation cohort: F.L.). Adjudicators were blinded to CMR data throughout.

The primary composite arrhythmic endpoint was SCD, ventricular fibrillation, or sustained ventricular tachycardia. SCD was defined as per American Heart Association criteria (a death that occurred unexpectedly, occurring within ≤60 minutes of symptom onset, following an unsuccessful resuscitation, or occurring when the patient was seen alive and was clinically stable ≤24 hours before death and without another identifiable cause of death). Ventricular fibrillation was defined as rapid—>300 beats/min (cycle length: ≤180 ms)—irregular ventricular rhythm with marked variability in QRS complex cycle length, morphology, and amplitude. Sustained ventricular tachycardia was deﬁned as a ventricular rhythm faster than 100 beats/min lasting at least 30 seconds or requiring termination because of hemodynamic instability or by antitachycardia pacing or shocks. Only appropriate shocks following sustained ventricular fibrillation or sustained ventricular tachycardia were considered in the arrhythmic endpoint. The secondary endpoint was the combined endpoint of total mortality, cardiac transplantation, or left ventricular assist device implantation. This endpoint was included to allow competing-risks analyses.

### Statistical analysis

Four broad questions were considered in statistical analyses. 1) Are MF measures superior to LVEF in arrhythmic risk stratification? 2) If so, are quantified measures of TF and GZF superior to MF_VA_ alone? 3) Which measure of TF and GZF should be used? 4) Should they be used alone or in combination?

Continuous variables are expressed as mean ± SD. Nonnormally distributed variables are expressed as median (Q1-Q3). Cumulative incidence curves and the log-rank test were used to assess cumulative survival. The proportionality assumption was tested by assessing Schoenfeld residuals and slopes in log-log plots. Fine and Gray proportional subdistribution hazard models and the cumulative incidence function were used to assess relative risks in competing-risks analyses. Death attributable to a cause other than a primary major arrhythmic event and without prior VF or sustained VT was used as the competing risk. Patients were censored at the time of the first event. Absolute TF and GZF mass were considered as both continuous and dichotomous variables. Thresholds for TF and GZF in the subgroup with MF_VA_ in the derivation cohort were derived using log-rank maximization and bootstrapped (1,000 replications) to estimate CIs. An LVEF cutoff of <35% was selected given that current guidelines use this cutoff in primary prevention ICD recommendations.^3^, ^4^, ^5^

In reclassification analyses, the incremental value of MF_VA_ over an LVEF of <35% and of quantified TF and GZF over MF_VA_ alone was assessed using category-free net reclassification improvement (NRI) (bootstrapped using 1,000 replications). Harrell C-statistics were obtained from cause-specific Cox regression models. Uno C-statistics were also derived to account for uncensored events. Decision curve analysis was used to evaluate the net benefit of an MF_VA_ and quantified MF measures in comparison to LVEF. A 2-sided *P* value of <0.05 was considered significant. Statistical analyses were undertaken using Stata version 15 (StataCorp) (“incrisk” package for reclassification indices, “stcrreg” for competing-risks analyses using Fine and Gray distributions, and “stdca” for decision curve analysis). The PROC PHREG procedure in the SAS statistical package (SAS Institute) was used to derive the Uno statistics. Differences between C-statistics were assessed using the “roccomp” command.

## Results

### Baseline characteristics

**Figure 2:**
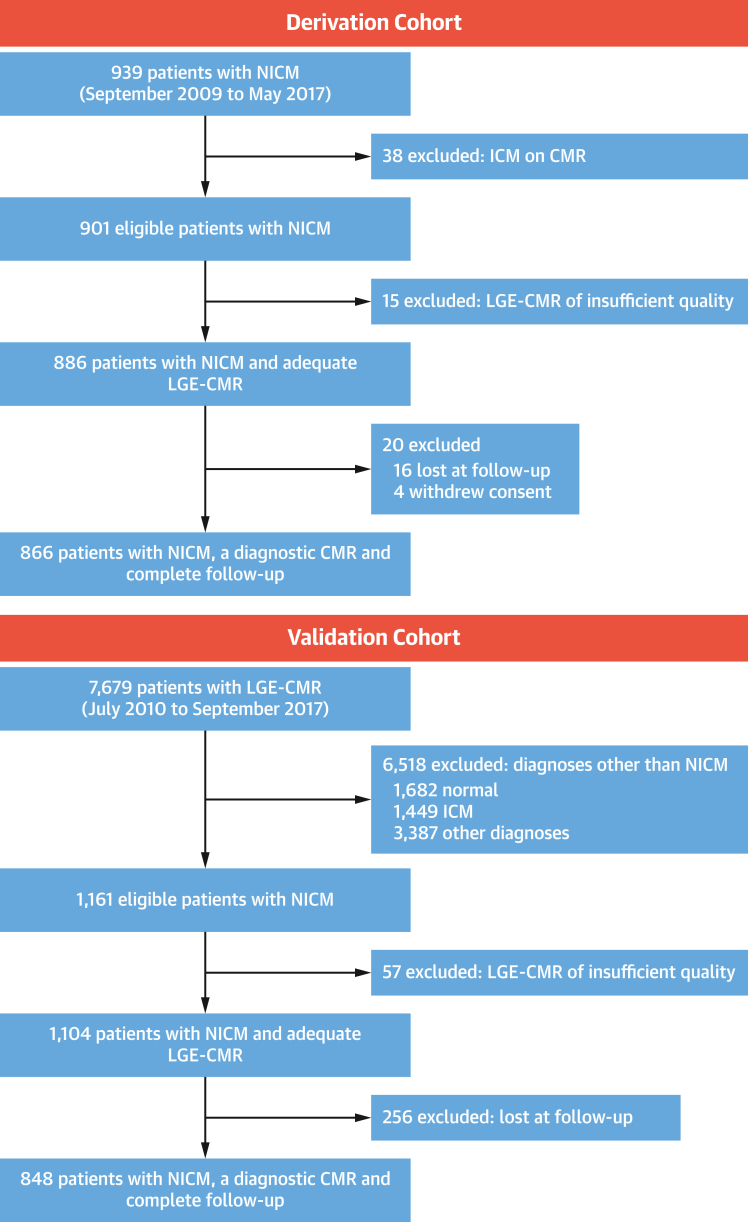
Study Flow Chart Flow chart illustrating the assembly of the derivation and validation cohorts. CMR = cardiovascular magnetic resonance; ICM = ischemic cardiomyopathy; LGE = late gadolinium enhancement; NICM = nonischemic cardiomyopathy.

The derivation cohort included 866 patients, prospectively enrolled from 2009 to 2017 (mean age 53.1 ± 14.9 years; 561 of 866 [64.8%] male; LVEF 41.8% ± 13.5%) and followed up for 7.60 years (Q1-Q3: 5.43-9.44 years) (Figure 2). The validation cohort included 848 patients, retrospectively enrolled from 2010 to 2017 (mean age 53.5 ± 16.9 years; 540 of 848 [63.7%] male; LVEF 45.3% ± 17.9%) and followed up for 6.81 years (Q1-Q3: 5.23-8.36 years) (Figure 2). In the derivation cohort, which mainly comprised patients who were not local to the hospital, postal questionnaire responses were returned by 590 of 865 (68%) patients. Complete follow-up data from either primary care records, hospital records, or postal questionnaires was available for most patients in the derivation cohort except the 16 who were excluded (Figure 2). In the validation cohort, which mainly comprised local patients, complete follow-up was available in all patients without the need for postal questionnaires.

As shown in Table 1, the derivation cohort had a higher proportion of patients with diabetes mellitus and hypertension and a lower proportion of patients with MF_VA_ (33.7% vs 56.6%; *P* < 0.001). Patients in the subgroup with MF_VA_ in the derivation cohort had a higher TF_2SD_ and GZF_3SD_ mass than those with MF_VA_ in the validation cohort (both *P* < 0.001) (Table 1). Further TF and GZF characteristics are shown in Supplemental Table 1.

**Table 1:** Baseline Characteristics

	Derivation Cohort (n = 866)	Validation Cohort (n = 848)	*P* Value
Age, y	53.1 ± 14.9	53.5 ± 16.9	0.567
Male	561 (64.8)	540 (63.7)	0.634
Diabetes mellitus	93 (10.8)	47 (5.54)	<0.001
Hypertension	256 (29.6)	147 (17.3)	<0.001
CMR volumes			
Absolute			
LVEDV, mL	241.9 ± 74.6	187.1 ± 78.2	<0.001
LVESV, mL	146.3 ± 73.8	110.4 ± 76.0	<0.001
LV mass, g	173.4 ± 56.0	154.9 ± 54.8	<0.001
LVEF, %	41.8 ± 13.5	45.3 ± 17.9	<0.001
LVEF <35%	272 (31.4)	253 (29.8)	0.480
LVEF >35%	594 (68.6)	595 (70.2)	
Indexed			
LVEDVi, mL/m^2^	121.5 ± 35.2	96.1 ± 39.5	<0.001
LVESVi, mL/m^2^	73.4 ± 36.0	56.8 ± 39.0	<0.001
LV mass index, g/m^2^	86.5 ± 24.5	79.5 ± 25.9	<0.001
MF_VA_	292 (33.7)	480 (56.6)	<0.001
MF pattern			
No MF	574 (66.3)	368 (43.4)	<0.001
Midwall	235 (27.1)	366 (43.2)	
Other	57 (6.58)	114 (13.4)	
Subgroup with MF_VA_	292 (33.7)	480 (56.6|)	<0.001
TF_2SD_ mass, g	8.51 (5.19-14.0)	4.07 (1.66-9.57)	<0.001
GZF_3SD_ mass, g	2.84 (1.67-4.24)	1.79 (0.79-3.58)	<0.001

Values are mean ± SD, n (%), or median (Q1-Q3).

CMR = cardiovascular magnetic resonance; GZF_3SD_ = gray zone fibrosis according to the 3-SD method; LV = left ventricular; LVEDV = left ventricular end-diastolic volume; LVEDVi = left ventricular end-diastolic volume index; LVEF = left ventricular ejection fraction; LVESV = left ventricular end-systolic volume; LVESVi = left ventricular end-systolic volume index; MF = myocardial fibrosis; MF_VA_ = myocardial fibrosis on visual assessment; TF_2SD_ = total fibrosis according to the 2-SD method.

In the derivation cohort, 52 of 866 (6.00%) patients met the primary endpoint over a median of 7.60 years (Q1-Q3: 5.43-9.44 years). Clinical events are listed in Table 2.

**Table 2:** Events

	Derivation Cohort (n = 866)	Validation Cohort (n = 848)
Sudden cardiac death, ventricular tachycardia, or ventricular fibrillation^a^	52 (6.00)	32 (3.77)
Sudden cardiac death	12 (1.39)	9 (1.30)
Ventricular tachycardia	31 (3.58)	17 (2.0)
Ventricular fibrillation	9 (1.04)	6 (0.71)
Total mortality, cardiac transplantation, or LVAD implantation	147 (16.97)	155 (18.3)
Total mortality	128 (14.78)	140 (16.5)
Cardiac transplantation	15 (1.73)	10 (1.18)
LVAD implantation	11 (1.27)	5 (0.59)
Unknown cause	6 (0.69)	10 (1.18)
Cardiac implantable electronic device implantation^b^		
All devices	241 (27.8)	207 (24.2)
Pacemaker	9 (1.04)	3 (0.35)
CRT-P	34 (3.92)	77 (9.08)
CRT-D	122 (14.09)	62 (7.31)
ICD	76 (8.78)	65 (7.67)

Values are n (%).

CRT-D = cardiac resynchronization therapy with defibrillation; CRT-P = cardiac resynchronization therapy–pacing; ICD = implantable cardioverter-defibrillator; LVAD = left ventricular assist device.

a Refers to patients meeting the primary endpoint of sudden cardiac death or ventricular tachycardia/ventricular fibrillation, whichever occurred first.

b Refers to devices implanted after the cardiovascular magnetic resonance scan.

### MF on Visual Assessment

**Figure 3:**
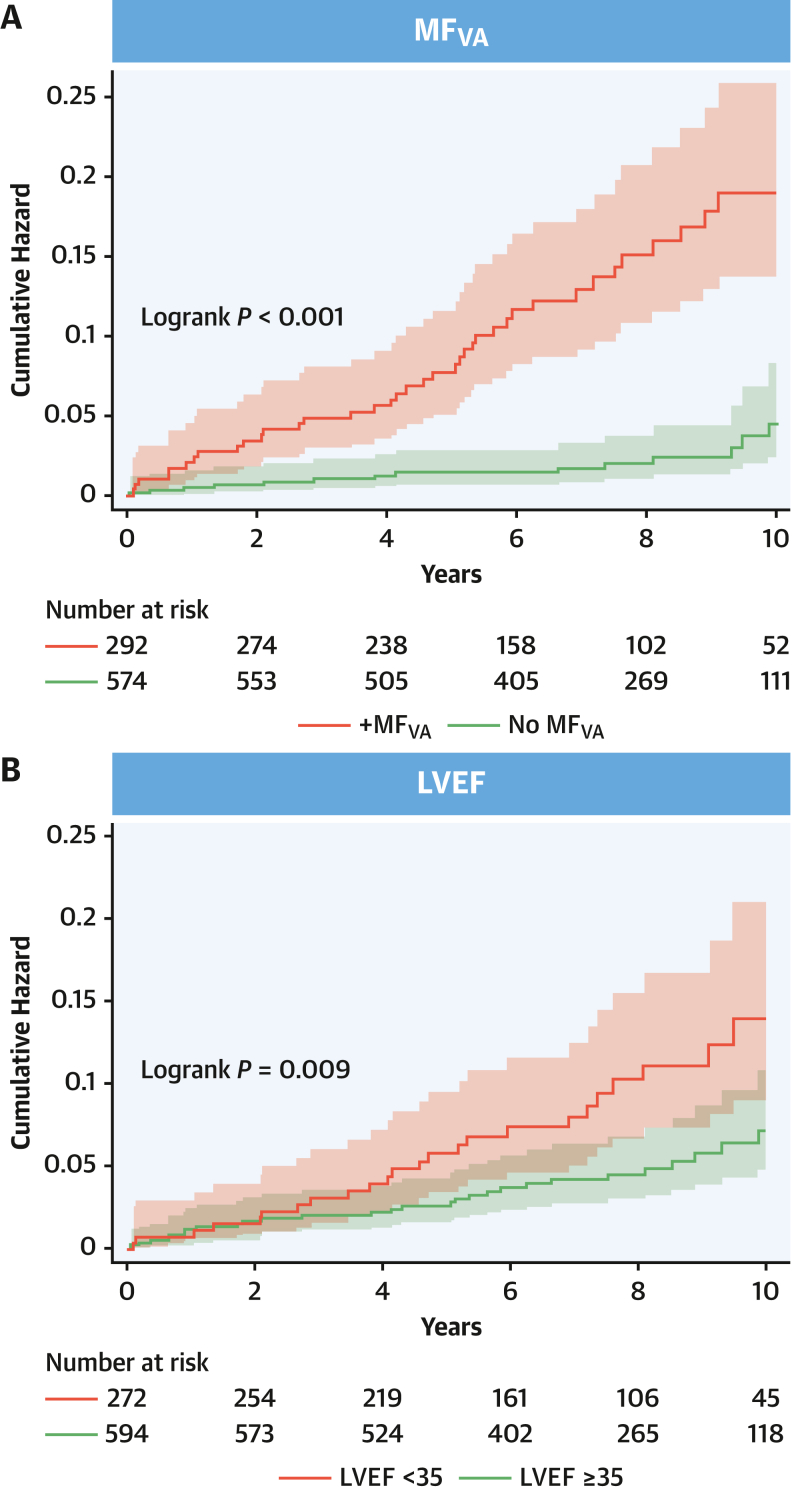
MF_VA_ and LVEF in Relation to the Primary Endpoint Cumulative hazard estimates for the primary endpoint in the derivation sample, stratified by (A) MF_VA_ or (B) LVEF (<35% or ≥35%). LVEF = left ventricular ejection fraction; MF_VA_ = myocardial fibrosis on visual assessment.

In the derivation cohort, both MF_VA_ (log-rank *P* < 0.001) and LVEF of <35% (log-rank *P* = 0.009) were associated with a higher cumulative incidence of the primary endpoint (Figure 3). MF_VA_ was associated with a C-statistic of 0.71 (95% CI: 0.65-0.77) (Table 3, Supplemental Table 2), a Harrell C-statistic of 0.72, and an Uno C-statistic of 0.68 (Supplemental Table 3). In competing-risks univariate analyses, MF_VA_ predicted the primary endpoint (HR: 5.83; 95% CI: 3.15-10.8; *P* < 0.001) (Table 4). A similar trend was observed in the validation cohort (Supplemental Table 4). In multivariable analyses, MF_VA_ predicted the primary endpoint after adjusting for LVEF of <35% (derivation cohort: HR: 5.52; 95% CI: 2.97-10.2; *P* < 0.001; validation cohort: HR: 3.87; 95% CI: 1.58-9.49; *P* = 0.003) (Table 5).

**Table 3:** Receiver-Operator Characteristic Analyses

	C-Statistic	Sensitivity, %	Specificity, %	PPV, %	NPV, %	PLR	NLR
Derivation cohort							
LVEF, %	0.63 (0.55-0.71)	46.2 (32.2-60.5)	69.5 (66.2-72.7)	8.82 (5.74-12.8)	95.3 (93.3-96.8)	1.51 (1.11-2.07)	0.77 (0.60-1.00)
LVEF <35%	0.58 (0.51-0.65)	46.2 (32.2-60.5)	69.5 (66.2-72.7)	8.82 (5.74-12.8)	95.3 (93.3-96.8)	1.51 (1.11-2.07)	0.77 (0.60-1.00)
MF_VA_	0.71 (0.65-0.77)	73.1 (59.0-84.4)	68.8 (65.5-72.0)	13.0 (9.38-17.4)	97.6 (95.9-98.7)	2.34 (1.93-2.84)	0.39 (0.25-0.61)
TF_2SD_, g	0.73 (0.66-0.80)	40.4 (27.0-54.9)	88.8 (86.5-90.9)	18.8 (12.0-27.2)	95.9 (94.2-97.2)	3.61 (2.46-5.30)	0.67 (0.54-0.84)
GZF_3SD_, g	0.73 (0.66-0.80)	48.1 (34.0-62.4)	86.5 (83.9-88.8)	18.5 (12.4-26.1)	96.3 (94.7-97.6)	3.56 (2.55-4.96)	0.60 (0.46-0.78)
Validation cohort							
LVEF, %	0.61 (0.51-0.72)	46.9 (29.1-65.3)	70.8 (67.6-73.9)	5.93 (3.40-9.60)	97.1 (95.5-98.3)	1.61 (1.09-2.36)	0.75 (0.54-1.04)
LVEF <35%	0.59 (0.50-0.68)	46.9 (29.1-65.3)	70.8 (67.6-73.9)	5.93 (3.40-9.60)	97.1 (95.5-98.3)	1.61 (1.09-2.36)	0.75 (0.54-1.04)
MF_VA_	0.63 (0.56-0.70)	81.3 (63.6-92.8)	44.4 (40.9-47.8)	5.42 (3.60-7.80)	98.4 (96.5-99.4)	1.46 (1.22-1.74)	0.42 (0.21-0.87)
TF_2SD_, g	0.67 (0.58-0.77)	25.0 (11.5-43.4)	87.6 (85.2-89.8)	7.34 (3.20-14.0)	96.8 (95.2-97.9)	2.02 (1.08-3.78)	0.86 (0.70-1.05)
GZF_3SD_, g	0.66 (0.57-0.75)	31.3 (16.1-50.0)	82.7 (79.9-85.3)	6.62 (3.20-11.8)	96.8 (95.3-98.0)	1.81 (1.06-3.09)	0.83 (0.66-1.05)

Values are area under the curve (95% CI). Shown are the results of receiver-operator characteristic analyses in the derivation cohort. For analysis of differences between C-statistics, please see Supplemental Table 2.

NLR = negative likelihood ratio; NPV = negative predictive value; PLR = positive likelihood ratio; PPV = positive predictive value; other abbreviations as in Table 1.

**Table 4:** Univariate Analysis

	Subdistribution HR (95% CI)	Annual Event Rate, %	*P* Value	Harrell C-Statistic^a^
LVEF				
Per %	0.97 (0.95-0.99)	—	0.002	0.63
≥35%	Reference	0.66	—	—
<35%^b^	1.91 (1.11-3.29)	1.33	0.02	0.58
MF_VA_				
No MF_VA_	Reference	0.34	—	—
MF_VA_ present	5.83 (3.15-10.8)	2.01	<0.001	0.72
TF_2SD_^c^				
Per gram	1.05 (1.04-1.07)	—	<0.001	0.75
>0 to ≤10 g	4.03 (1.99-8.16)	1.38	<0.001	—
>10 g	9.17 (4.64-18.1)	3.15	<0.001	0.74
GZ_3SD_^c^				
Per gram	1.16 (1.11-1.22)	—	<0.001	0.75
>0 to ≤3 g	3.53 (7.50-1.21)	1.21	0.001	—
>3 g	8.84 (4.59-17.0)	3.05	<0.001	0.75

Shown are results from competing-risks analyses for the derivation cohort. The event rates refer to annual event rates for the primary endpoint.

Abbreviations as in Table 1.

a Harrell C-statistics were derived from Cox regression analyses.

b Compared to LVEF of ≥35%.

c Categories are compared to no MF_VA_.

**Table 5:** Multivariable Analyses

	Subdistribution HR (95% CI)	*P* Value	Harrell C-Statistic^a^
Model 1			0.74
MF_VA_	5.52 (2.97-10.2)	<0.001	
LVEF <35%	1.52 (0.88-2.64)	0.132	
Model 2			0.73
TF_2SD_, g	1.05 (1.03-1.07)	<0.001	
LVEF, %	0.97 (0.95-0.99)	0.018	
Model 3			0.72
GZ_3SD_, g	1.14 (1.08-1.20)	<0.001	
LVEF, %	0.97 (0.95-0.99)	0.019	

Shown are results from competing-risks analyses for the derivation cohort.

Abbreviations as in Table 1.

a Harrell C-statistics were derived from Cox regression analyses.

In risk category net reclassification analyses of the derivation sample, the addition of MF_VA_ to a predictive model containing LVEF of <35% alone resulted in a continuous NRI of 0.84 (95% CI: 0.58-1.06) (Supplemental Table 5).

### TF and GZF mass

Having explored the utility of MF_VA_, further analyses focused on the predictive value of quantified TF and GZF mass. On the basis of univariate analyses (Supplemental Table 6), TF_2SD_ and GZF_3SD_ emerged as the most consistent predictors of the primary endpoint across the derivation and validation cohorts.

As shown in Table 3 and Supplemental Table 2, C-statistics were 0.73 (95% CI: 0.66-0.80) for both TF_2SD_ and GZF_3SD_. The Harrell (0.75) and Uno (0.70) C-statistics were identical (Supplemental Table 3). Optimal cutoffs, derived from the derivation cohort subgroup with MF_VA_, were 9.99 g and 3.16 g for TF_2SD_ and for GZF_3SD_, respectively. As shown in Table 4, TF_2SD_ and GZF_3SD_ mass, according to these cutoffs, permitted categorization into 3 risk groups. In the derivation cohort, a TF_2SD_ of >0 g and ≤10 g was associated with an intermediate risk of the primary endpoint (HR: 4.03; 95% CI: 1.99-8.16), and a TF_2SD_ of >10 g was associated with the highest risk (HR: 9.17; 95% CI: 4.64-18.1) compared to patients with no MF_VA_. Similarly, a GZF_3SD_ of >0 and ≤3 g was associated with a medium risk of the primary endpoint (HR: 3.53; 95% CI: 1.66-7.50), and a GZF_3SD_ of >3 g was associated with the highest risk (HR: 8.84; 95% CI: 4.59-17.0). A similar trend was observed in the validation cohort (Supplemental Table 6).

**Figure 4:**
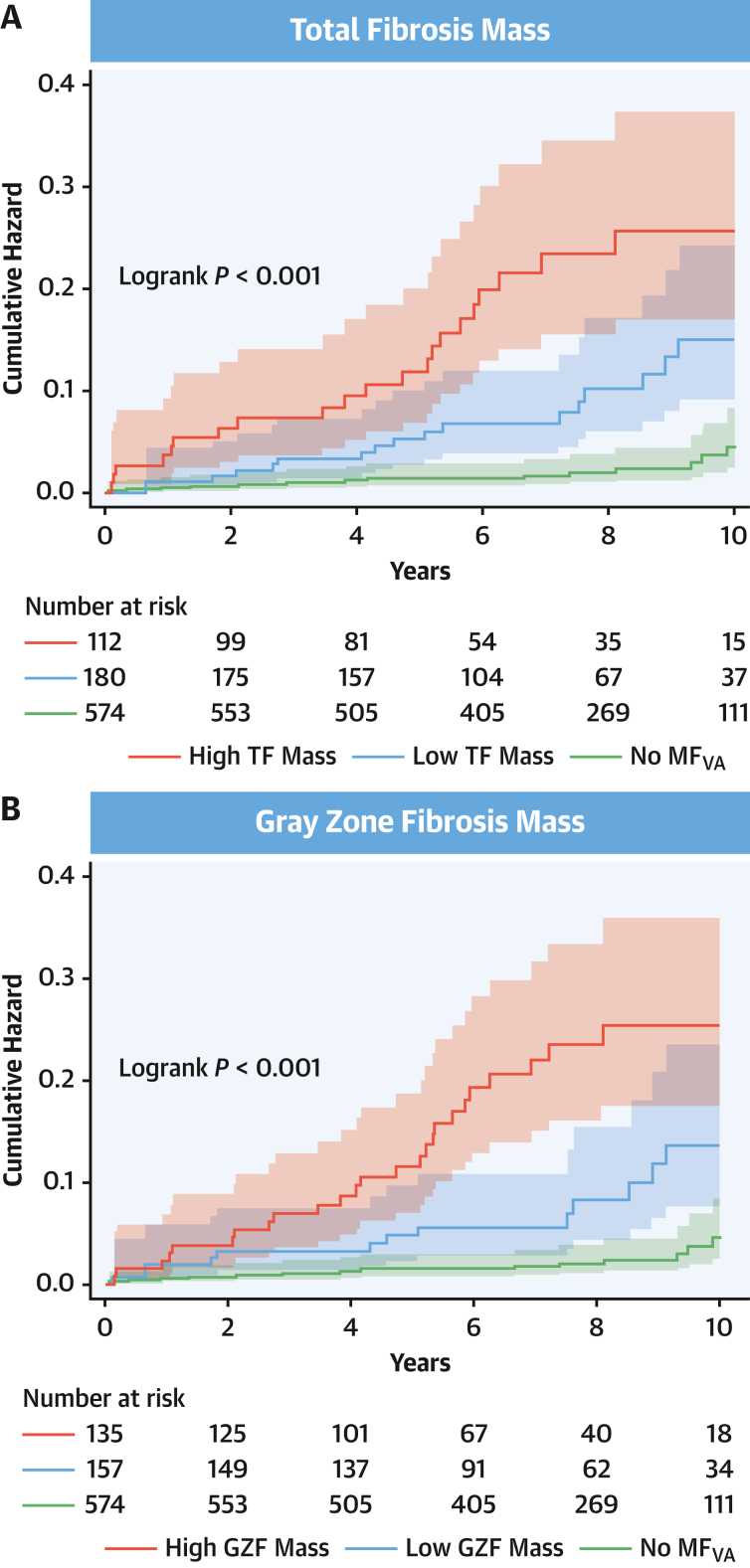
Quantified TF and GZF in Relation to the Primary Endpoint Cumulative hazard estimates of the primary arrhythmic endpoint in the derivation sample, stratified according to (A) total fibrosis mass according to the 2-SD method (low: >0 and ≤10 g; high: >10 g) and (B) gray zone fibrosis mass according to the 3-SD method (low: >0 and ≤3 g; high: >3 g). GZF = gray zone fibrosis; MF_VA_ = myocardial fibrosis on visual assessment; TF = total fibrosis.

As shown in Figure 4, categorization into low-, intermediate-, and high-risk groups was achievable using either TF_2SD_ or GZF_3SD_ mass. In the derivation sample, annual event rates for the primary endpoint were 1.38% for TF_2SD_ of >0 and ≤10 g and 3.15% for TF_2SD_ of >10 g (Table 3). Corresponding event rates for GZF_3SD_ were 1.21% for GZF_3SD_ of >0 and ≤3 g and 3.05% for GZF_3SD_ of >3 g). The lowest event rates were observed in patients with no MF_VA_ (0.34%). The combination of both TF_2SD_ or GZF_3SD_ also permitted categorization into 3 risk groups (Supplemental Figure 1).

In net reclassification analyses of the derivation sample, the addition of quantified TF_2SD_ to MF_VA_ resulted in a category-free NRI of 0.17 (95% CI: –0.22 to 0.42) (Supplemental Table 7). The addition of GZF_3SD_ to MF_VA_ was associated with an NRI of 0.27 (95% CI: 0.05-0.51) (Supplemental Table 8).

### LV ejection fraction

**Figure 5:**
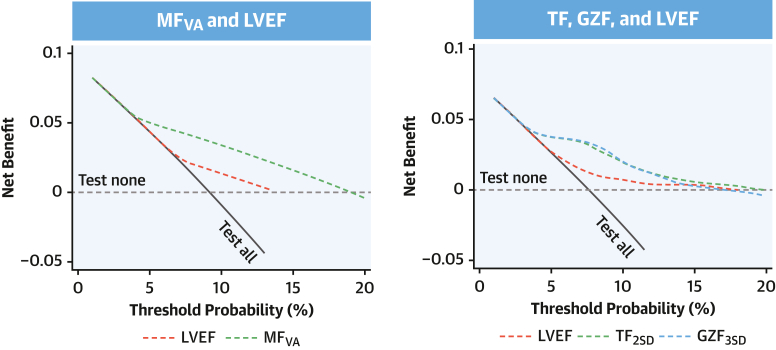
Decision Curve Analysis The graphs show decision curves in the derivation cohort, comparing the net benefit of MF (*y*-axis) across different threshold probabilities of the primary endpoint (*x*-axis). The decision curve reflects the tradeoff between true positive predictions and false positive predictions for a given strategy. The area under the decision curve quantifies the overall clinical utility of the predictive model. The dotted horizontal gray line indicates the net benefit of not testing any patient (“test none”), whereas the solid diagonal line shows the net benefit of testing all patients (“test all”). The dashed colored decision curves indicate the net benefit of using LVEF or MF measures in prediction models. See Supplemental Figure 1 for analysis of the validation cohort. GZF = gray zone fibrosis; GZF_3SD_ = gray zone fibrosis according to the 3-SD method; LVEF = left ventricular ejection fraction; MF_VA_ = myocardial fibrosis on visual assessment; TF = total fibrosis; TF_2SD_ = total fibrosis according to the 2-SD method.

In the derivation cohort, LVEF of <35% was associated with the primary endpoint on univariate analysis (HR: 1.91; 95% CI: 1.11-3.29) but failed to reach significance in a multivariable model when MF_VA_ was added as a covariable (HR: 1.52; 95% CI: 0.88-2.64). The C-statistic for LVEF of <35% was 0.58 (95% CI: 0.51-0.65) in the derivation cohort. In the validation cohort, LVEF of <35% was not associated with the primary endpoint on univariate analysis (HR: 1.99; 95% CI: 0.99-4.01; *P* = 0.053) but was only associated with the primary endpoint in a multivariable model that included MF_VA_ (HR: 2.32; 95% CI: 1.14-4.73; *P* = 0.021). In decision curve analyses (Figure 5, Supplemental Figure 2), MF_VA_ as well as quantified MF measures were superior to LVEF in predicting the primary endpoint.

## Discussion

This is the largest study of CMR-derived measures of MF in relation to SCD and VAs in patients with NICM. Unique aspects are external validation and the inclusion of patients with any LVEF. Several findings have emerged (Central Illustration). First, MF_VA_ was a powerful predictor of the primary endpoint of SCD and VAs. Second, quantification of TF_2SD_ and GZF_3SD_ mass were of incremental value to using MF_VA_ alone, permitting further risk stratification into low-, intermediate-, and high-risk categories. Third, this risk categorization was achievable using TF_2SD_ and GZF_3SD_ mass in isolation or in combination. Last, LVEF was a poor predictor of SCD and VAs.

**Central Illustration:**
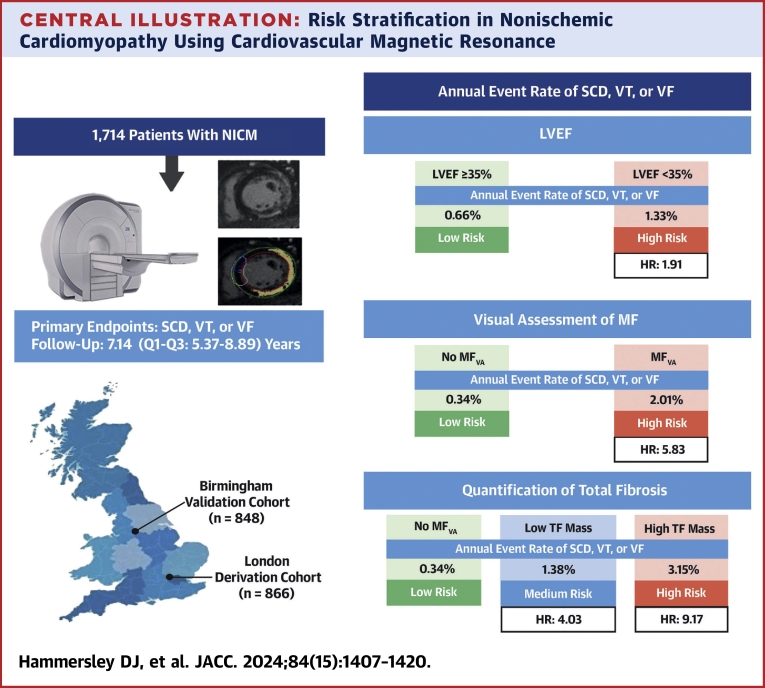
Risk Stratification in Nonischemic Cardiomyopathy Using Cardiovascular Magnetic Resonance A total of 1,714 patients with (NICM) were enrolled independently in 2 centers: a derivation cohort and a validation cohort. Late gadolinium enhancement cardiovascular magnetic resonance was used to determine the presence of MF_VA_. If MF_VA_ was present, TF and gray zone fibrosis was quantified. To this end, areas of MF, which appear white on late gadolinium enhancement, were semiautomatically delineated on short-axis images, using signal thresholding techniques. As shown at the upper right, LVEF of <35% was associated with a higher risk of the primary endpoint on competing-risks analyses (HR: 1.91; 95% CI: 1.11-3.29) of the derivation cohort. MF_VA_ was a powerful predictor of the primary endpoint (HR: 5.83; 95% CI: 3.15-10.8) (middle right). Quantification of TF permitted categorization into low-, intermediate- (HR: 4.03; 95% CI: 1.99-8.16), and high-risk (HR: 9.17; 95% CI: 4.64-18.1) groups (bottom right). TF mass was quantified according to the 2-SD method and expressed as low (>0 to ≤10 g) or high (>10 g). LGE = late gadolinium enhancement; LVEF = left ventricular ejection fraction; MF = myocardial fibrosis; MF_VA_ = myocardial fibrosis on visual assessment; NICM = nonischemic cardiomyopathy; SCD = sudden cardiac death; TF = total fibrosis; VF = ventricular fibrillation; VT = ventricular tachycardia.

### MF on Visual Assessment

We, as others,^16^, ^17^, ^18^, ^19^, ^20^, ^21^, ^22^, ^23^ have found that MF_VA_ is associated with a high risk of arrhythmic events compared with no MF_VA_. The level of risk was 5.83-fold higher, which is similar to that reported by 4 large meta-analyses using a similar endpoint (ORs of 3.99 in Theerasuwipakorn et al,^19^ 3.99 in Di Marco et al,^18^ 4.52 in Becker et al,^21^ and 5.62 in Disertori et al^23^). In our derivation cohort, this risk equates to annualized event rates of 2.01% for MF_VA_ and 0.34% for no MF_VA_. The C-statistic for MF_VA_ of 0.71 was associated with a low positive predictive value of 13% but a high negative predictive value of 98%. In other words, absence of MF_VA_ virtually excluded SCD and VAs in the long term, regardless of LVEF. Remarkably, the category-free NRI for MF_VA_ compared with LVEF of <35% was 84%.

### TF and GZF mass

Because dense MF and GZF form part of the arrhythmogenic substrate in both ICM and NICM,^15^ one would expect a higher TF and GZF mass to carry a higher arrhythmic risk. In this regard, few CMR studies in the field of arrhythmic risk stratification in NICM have used formal quantification of MF. Semiquantitative assessment, however, has been undertaken by several large multicenter studies. In the DERIVATE (Cardiac Magnetic Resonance for Prophylactic Implantable-Cardioverter Defibrillator Therapy in Non-Ischaemic Dilated Cardiomyopathy) registry of 1,508 patients with NICM, LV midwall fibrosis in >3 myocardial segments emerged as a predictor of major adverse arrhythmic events.^31^ Using a similar semiquantitative approach in 1,615 patients with dilated cardiomyopathy and an LVEF of <45%, Di Marco et al^18^ found that combining MF and 3 LVEF strata was superior to LVEF of <35% in risk stratification (SCD and VAs; Harrell C-statistic: 0.80 vs 0.69, respectively).^18^ Similar findings were reported by Klem et al^6^ in a prospective registry of 1,020 NICM patients.

We found that TF_2SD_ and GZF_3SD_ mass permitted stratification of arrhythmic risk into low-, intermediate- and high-risk categories, corresponding to annualized event rates of 0.34%, 1.38%, and 3.15%, respectively (using TF_2SD_ in the derivation cohort). The NRI for TF_2SD_ and GZF_3SD_ mass over MF_VA_ were 17% and 27%, respectively, indicating that quantification of TF_2SD_ and GZF_3SD_ mass has incremental value in arrhythmic risk stratification, albeit modest. Importantly, using the combination of TF_2SD_ and GZF_3SD_ mass had no incremental value over TF_2SD_ or GZF_3SD_ in isolation. In the interest of simplicity, therefore, only TF_2SD_ or GZF_3SD_ mass, but not both, need to be quantified.

### LV Ejection Fraction

We found that LVEF was not associated with the primary endpoint on univariate analysis in the validation cohort. In the derivation cohort, it failed to reach significance as a predictor of the primary endpoint when MF_VA_ was added as a covariable, suggesting that LVEF is perhaps a surrogate of myocardial scar. In essence, LVEF was not a reliable predictor of the primary endpoint on external validation across both cohorts. These findings are consistent with those of Klem et al,^6^ who found no association between LVEF of ≤35% and SCD in 1,020 NICM patients. Overall, it is not surprising that LVEF is a poor predictor of VAs. After all, it is a measure of cardiac contraction rather than the arrhythmic substrate.

### Clinical application

Our findings indicate that arrhythmic risk stratification should be based on characterization of the arrhythmic substrate rather than on LVEF. They are broadly consistent with previous CMR studies showing that in patients with ICM^26^^,^^27^ and with cardiac implantable electronic devices,^30^ MF is better than LVEF at predicting arrhythmic events. We have shown that not all NICMs have the same arrhythmogenic potential: some patients with MF_VA_ are at a high risk of SCD and VAs (>3% per year, or >15% in 5 years), whereas those with no MF_VA_ are at low risk (0.34% per year), regardless of LVEF. Although the present study does not address the benefits of ICD therapy, our findings support the use of MF measures rather than LVEF in making decisions on ICD implantation for primary prevention. The strongest suggestion is that the low annual event rate in NICM patients with no MF_VA_ may not justify the use of primary prevention ICDs. Ongoing randomized controlled trials are addressing these issues.^32^^,^^33^

### Study limitations

Although the inclusion of a retrospective cohort may be considered a limitation, it is arguably a strength insofar as the validation exercise focuses on real-world practice. By this token, there are differences in baseline characteristics. Importantly, this study focuses on a single CMR scan. In this regard, we should consider that NICM (as opposed to ICM) involves a chronic inflammatory process^34^^,^^35^ and that patients who did not have MF_VA_ at the outset may have developed it thereafter. The role of serial risk stratification using CMR requires further scrutiny. Although we have excluded asymptomatic and nonsustained VAs, we should consider that arrhythmias are more likely to be detected in patients with implanted cardiac devices and that not all VAs detected by cardiac implantable electronic devices are clinically meaningful.

## Conclusions

In this large study of patients NICM, MF_VA_ was a powerful predictor of the primary arrhythmic endpoint of SCD and VAs. Quantification of both TF_2SD_ and GZF_3SD_ mass added value to using MF_VA_ alone, permitting classification into low-, intermediate-, and high-risk groups. In contrast, LVEF was a poor predictor. These findings support the approach of using MF_VA_ and quantifying either TF_2SD_ or GZF_3SD_ for the arrhythmic risk stratification of patients with NICM. Randomized controlled trials are required to address whether such measures should be used in preference to LVEF in selecting patients for primary prevention ICDs.

Perspectives **COMPETENCY IN PATIENT CARE AND PROCEDURAL SKILLS:** MF visually assessed from CMR imaging is a powerful predictor of ventricular arrhythmias and sudden cardiac death in patients with NICM, whereas LVEF was a relatively poor predictor of these events.

**TRANSLATIONAL OUTLOOK:** Prospective studies are needed to determine how best to incorporate estimates of MF severity based on CMR in the selection of patients for primary prevention implanted cardiac defibrillators.

## Funding Support and Author Disclosures

This work was supported by a National Heart and Lung Institute Foundation grant awarded to Drs Prasad, Hammersley, Jones, Tayal, and Halliday as well as a British Society for Heart Failure Research Fellowship and a British Heart Foundation Clinical Research Training Fellowship (FS/CRTF/23/24444) awarded to Dr Mach. Additionally, the study was supported by Rosetrees Trust, the Alexander Jansons Myocarditis UK Foundation, a BHF Intermediate Clinical Research Fellowship awarded to Dr Halliday (FS/ICRF/21/26019), and an MRC Fellowship awarded to Dr Tayal (MRC MR/W023830/1). This work was additionally supported by The British Heart Foundation (RE/18/4/34215; SP/17/11/32885), Royston Centre for Cardiomyopathy Research, Sir Jules Thorn Charitable Trust (21JTA), Medical Research Council (UK), National Institute for Health Research, Royal Brompton Cardiovascular Biomedical Research Unit, and National Institute for Health Research Imperial College Biomedical Research Centre. Medtronic Plc provided funding for the salary as a research fellow for Dr Zegard. Boston Scientific provided funding for Dr Qiu (statistician). These companies had no participation whatsoever in the study. The views expressed in this work are those of the authors and not necessarily those of the funders. Dr Hammersley has received research funding from Siemens. Dr Baruah is an employee of AstraZeneca. Dr Guha has received honoraria from Bayer, Pfizer, Novartis, AstraZeneca, and Servier Laboratories; has received an unrestricted educational grant from Biotronik; and has received travel assistance from Abbott Laboratories, Medtronic, Biotronik, and Boston Scientific. Dr Ware has acted as a consultant for MyoKardia, Foresite Labs, Pfizer, and Health Lumen. Dr Halliday has received honoraria from AstraZeneca. All other authors have reported that they have no relationships relevant to the contents of this paper to disclose.

## References

[1] Arbelo E., Protonotarios A., Gimeno J.R. (2023). 2023 ESC guidelines for the management of cardiomyopathies. Eur Heart J.

[2] Chrispin J., Merchant F.M., Lakdawala N.K. (2023). Risk of arrhythmic death in patients with nonischemic cardiomyopathy: *JACC* review topic of the week. J Am Coll Cardiol.

[3] Heidenreich P.A., Bozkurt B., Aguilar D. (2022). 2022 AHA/ACC/HFSA guideline for the management of heart failure. J Am Coll Cardiol.

[4] McDonagh T.A., Metra M., Adamo M. (2021). 2021 ESC guidelines for the diagnosis and treatment of acute and chronic heart failure: developed by the Task Force for the Diagnosis and Treatment of Acute and Chronic Heart Failure of the European Society of Cardiology (ESC). With the special contribution of the Heart Failure Association (HFA) of the ESC. Eur Heart J.

[5] Zeppenfeld K., Tfelt-Hansen J., de Riva M. (2022). 2022 ESC guidelines for the management of patients with ventricular arrhythmias and the prevention of sudden cardiac death: developed by the Task Force for the Management of Patients With Ventricular Arrhythmias and the Prevention of Sudden Cardiac Death of the European Society of Cardiology (ESC). Endorsed by the Association for European Paediatric and Congenital Cardiology (AEPC). Eur Heart J.

[6] Klem I., Klein M., Khan M. (2021). Relationship of LVEF and myocardial scar to long-term mortality risk and mode of death in patients with nonischemic cardiomyopathy. Circulation.

[7] Marijon E., Garcia R., Narayanan K., Karam N., Jouven X. (2022). Fighting against sudden cardiac death: need for a paradigm shift—adding near-term prevention and pre-emptive action to long-term prevention. Eur Heart J.

[8] Ramakrishna S., Salazar J.W., Olgin J.E., Moffatt E., Tseng Z.H. (2023). Heart failure burden by autopsy, guideline-directed medical therapy, and ICD utilization among sudden deaths. JACC Clin Electrophysiol.

[9] Gorgels A.P., Gijsbers C., de Vreede-Swagemakers J., Lousberg A., Wellens H.J. (2003). Out-of-hospital cardiac arrest—the relevance of heart failure. The Maastricht Circulatory Arrest Registry. Eur Heart J.

[10] Poole J.E., Olshansky B., Mark D.B. (2020). Long-term outcomes of implantable cardioverter-defibrillator therapy in the SCD-HeFT. J Am Coll Cardiol.

[11] Kober L., Thune J.J., Nielsen J.C. (2016). Defibrillator implantation in patients with nonischemic systolic heart failure. N Engl J Med.

[12] Fishman G.I., Chugh S.S., DiMarco J.P. (2010). Sudden cardiac death prediction and prevention report from a National Heart, Lung, and Blood Institute and Heart Rhythm Society Workshop. Circulation.

[13] Sánchez-Somonte P., Quinto L., Garre P. (2021). Scar channels in cardiac magnetic resonance to predict appropriate therapies in primary prevention. Heart Rhythm.

[14] Liuba I., Muser D., Chahal A. (2021). Substrate characterization and outcome of catheter ablation of ventricular tachycardia in patients with nonischemic cardiomyopathy and isolated epicardial scar. Circ Arrhythm Electrophysiol.

[15] Piers S.R., Tao Q., de Riva Silva M. (2014). CMR-based identification of critical isthmus sites of ischemic and nonischemic ventricular tachycardia. JACC Cardiovasc Imaging.

[16] Gulati A., Jabbour A., Ismail T.F., Guha K., Khwaja J., Raza S. (2013). Association of fibrosis with mortality and sudden cardiac death in patients with nonischemic dilated cardiomyopathy. JAMA.

[17] Di Marco A., Anguera I., Schmitt M. (2017). Late gadolinium enhancement and the risk for ventricular arrhythmias or sudden death in dilated cardiomyopathy: systematic review and meta-analysis. JACC Heart Fail.

[18] Di Marco A., Brown P.F., Bradley J. (2021). Improved risk stratification for ventricular arrhythmias and sudden death in patients with nonischemic dilated cardiomyopathy. J Am Coll Cardiol.

[19] Theerasuwipakorn N., Chokesuwattanaskul R., Phannajit J. (2023). Impact of late gadolinium-enhanced cardiac MRI on arrhythmic and mortality outcomes in nonischemic dilated cardiomyopathy: updated systematic review and meta-analysis. Sci Rep.

[20] Kuruvilla S., Adenaw N., Katwal A.B., Lipinski M.J., Kramer C.M., Salerno M. (2014). Late gadolinium enhancement on cardiac magnetic resonance predicts adverse cardiovascular outcomes in nonischemic cardiomyopathy: a systematic review and meta-analysis. Circ Cardiovasc Imaging.

[21] Becker M.A.J., Cornel J.H., van de Ven P.M., van Rossum A.C., Allaart C.P., Germans T. (2018). The prognostic value of late gadolinium-enhanced cardiac magnetic resonance imaging in nonischemic dilated cardiomyopathy: a review and meta-analysis. JACC Cardiovasc Imaging.

[22] Alba A.C., Gaztañaga J., Foroutan F. (2020). Prognostic value of late gadolinium enhancement for the prediction of cardiovascular outcomes in dilated cardiomyopathy: an international, multi-institutional study of the MINICOR group. Circ Cardiovasc Imaging.

[23] Disertori M., Rigoni M., Pace N. (2016). Myocardial fibrosis assessment by LGE is a powerful predictor of ventricular tachyarrhythmias in ischemic and nonischemic LV dysfunction: a meta-analysis. JACC: Cardiovasc Imaging.

[24] Roes S.D., Borleffs C.J., van der Geest R.J. (2009). Infarct tissue heterogeneity assessed with contrast-enhanced MRI predicts spontaneous ventricular arrhythmia in patients with ischemic cardiomyopathy and implantable cardioverter-defibrillator. Circ Cardiovasc Imaging.

[25] Acosta J., Fernandez-Armenta J., Borras R. (2018). Scar characterization to predict life-threatening arrhythmic events and sudden cardiac death in patients with cardiac resynchronization therapy: the GAUDI-CRT study. JACC Cardiovasc Imaging.

[26] Zegard A., Okafor O., Bono J.D. (2021). Myocardial fibrosis as a predictor of sudden death in patients with coronary artery disease. J Am Coll Cardiol.

[27] Jones R.E., Zaidi H.A., Hammersley D.J. (2023). Comprehensive phenotypic characterization of late gadolinium enhancement predicts sudden cardiac death in coronary artery disease. JACC Cardiovasc Imaging.

[28] Halliday B.P., Baksi A.J., Gulati A. (2019). Outcome in dilated cardiomyopathy related to the extent, location, and pattern of late gadolinium enhancement. JACC Cardiovasc Imaging.

[29] Halliday B.P., Gulati A., Ali A. (2017). Association between midwall late gadolinium enhancement and sudden cardiac death in patients with dilated cardiomyopathy and mild and moderate left ventricular systolic dysfunction. Circulation.

[30] Leyva F., Zegard A., Okafor O. (2022). Myocardial fibrosis predicts ventricular arrhythmias and sudden death after cardiac electronic device implantation. J Am Coll Cardiol.

[31] Guaricci A.I., Masci P.G., Muscogiuri G. (2021). Cardiac Magnetic Resonance for Prophylactic Implantable-Cardioverter Defibrillator Therapy in Nonischaemic Dilated Cardiomyopathy: an international registry. Europace.

[32] Flett A., Cebula A., Nicholas Z. (2023). Rationale and study protocol for the BRITISH randomized trial. (Using cardiovascular magnetic resonance identified scar as the benchmark risk indication tool for implantable cardioverter defibrillators in patients with nonischemic cardiomyopathy and severe systolic heart failure). Am Heart J.

[33] Selvanayagam J.B., Hartshorne T., Billot L. (2017). Cardiovascular magnetic resonance-guided management of mild to moderate left ventricular systolic dysfunction (CMR GUIDE): study protocol for a randomized controlled trial. Ann Noninvasive Electrocardiol.

[34] Mueller K.A., Heck C., Heinzmann D. (2016). Comparison of ventricular inducibility with late gadolinium enhancement and myocardial inflammation in endomyocardial biopsy in patients with dilated cardiomyopathy. PLoS One.

[35] Zaman S., Narayan A., Thiagalingam A. (2014). Long-term arrhythmia-free survival in patients with severe left ventricular dysfunction and no inducible ventricular tachycardia after myocardial infarction. Circulation.

