# Supplementary material for: Arrhythmic Risk Stratification by Cardiovascular Magnetic Resonance Imaging in Patients With Nonischemic Cardiomyopathy
Source: J Am Coll Cardiol. 2024 Oct 8;84(15):1407–20. doi: 10.1016/j.jacc.2024.06.046 (PMC11444937; doi:10.1016/j.jacc.2024.06.046)
Supplement: Supplementary Tables 1-8 and Supplementary Figures 1 and 2 [file mmc1.pdf]

Cardiovascular Magnetic Resonance for Arrhythmic Risk Stratification in Non-Ischemic Cardiomyopathy

SUPPLEMENTARY APPENDIX

CONTENTS

Page

2. **Table S1. Myocardial Fibrosis Measures Using Different Signal Threshold Methods.**

2. **Table S2. Univariate and Multivariable Analyses.**

3. **Table S3: Myocardial Fibrosis Measures in Relation to the Primary Endpoint in Both Cohorts.**

4. **Table S4. Comparison of C Statistics.**

4. **Table S5. Harrel C Statistics and Uno's C Statistics in the Derivation Cohort.**

5. **Table S6: Risk Reclassification Analyses: MF$_{VA}$ over LVEF.**

6. **Table S7: Risk reclassification ana Risk Reclassification Analyses: TF2SD mass over MFVA.**

7. **Table S8: Risk Reclassification Analyses: GZF$_{3SD}$ mass over MF$_{VA}$.**

8. **Figure S1. Quantified TF and GZF in Relation to the Primary Endpoint.**

9. **Figure S2. Decision Curve Analysis**

Table S1. Myocardial Fibrosis Measures Using Different Signal Threshold Methods.

	Derivation Cohort	Validation Cohort	p
**N**	**292**	**480**	
TF_{FWHM}, g	6.56 (3.60-11.3)	3.49 (1.37-7.39)	<0.001
TF_{2SD}, g	8.51 (5.19-14.0)	4.07 (1.66-9.57)	<0.001
TF_{3SD}, g	5.59 (3.18-9.51)	2.18 (0.80-5.55)	<0.001
TF_{5SD}, g	2.05 (1.11-3.44)	0.61 (0.13-1.72)	<0.001
GZF_{FWHM}, g	2.27 (1.02-4.36)	1.15 (0.17-3.20)	<0.001
GZF_{3SD}, g	2.84 (1.67-4.24)	1.79 (0.79-3.58)	<0.001
GZF_{5SD}, g	6.13 (3.80-9.86)	3.41 (1.45-7.33)	<0.001

Myocardial fibrosis measures using 2-, 3- and 5 standard deviation (SD) signal threshold methods in patients with myocardial fibrosis present on visual assessment, in derivation and validation cohorts. All values are expressed as median (interquartile range), in grammes.

FWHM = full width half maximum; GZF = gray zone fibrosis; TF = total fibrosis.

Table S2. Univariate and Multivariable Analyses.

	HR	95% CI		p
**Derivation Cohort**				
**Univariate**				
MF_{VA} present	5.83	3.15	10.8	<0.001
LVEF<35%	1.91	1.11	3.29	0.020
**Multivariable**				
MF_{VA} present	5.52	2.97	10.2	<0.001
LVEF<35%	1.52	0.88	2.64	0.132
**Validation Cohort**				
**Univariate**				
MF_{VA} present	3.49	1.44	8.45	0.006
LVEF<35%	1.99	0.99	4.01	0.053
**Multivariable**				
MF_{VA} present	3.87	1.58	9.49	0.003
LVEF<35%	2.32	1.14	4.73	0.021

Data presented as subdistribution hazard ratios with 95% confidence intervals (CI) and p values from competing risk Cox proportional hazard models.

Table S3: Myocardial Fibrosis Measures in Relation to the Primary Endpoint in Both Cohorts.

	**Derivation Cohort (N=866)**				**Validation Cohort (N=848)**			
	**SHR**	**95% C.I.**		**p**	**SHR**	**95% C.I.**		**p**
**TF measures**								
TF_{FWHM}	1.05	1.02	1.08	<0.001	1.05	1.02	1.08	<0.001
TF_{2SD}	1.05	1.04	1.07	<0.001	1.02	1.01	1.04	0.001
TF_{3SD}	1.03	1.01	1.04	0.002	1.03	1.01	1.04	0.002
TF_{5SD}	1.03	1.01	1.06	0.013	1.03	1.01	1.06	0.013
**GZF measures**								
GZF_{FWHM}	1.02	0.99	1.05	0.125	1.02	0.99	1.05	0.125
GZF_{3SD}	1.16	1.11	1.22	<0.001	1.10	1.03	1.17	0.002
GZF_{5SD}	1.05	1.02	1.07	0.001	1.05	1.02	1.07	0.001

Data presented as subdistribution hazard ratios with 95% confidence intervals (CI) and p values from competing risk Cox proportional hazard models. For myocardial fibrosis (MF) measures, patients without MF_{VA} on visual assessment were used as reference.

GZF = gray zone fibrosis; TF = total fibrosis; FWHM = full width, half-maximum. The other subscripts refer to the method used to in quantification, in terms of standard deviations.

Table S4. Comparison of C Statistics.

	LVEF < 35%	LVEF (%)
**Derivation Cohort**		
MF_{VA}	0.004	-
TF_{2SD}, g	-	0.037
GZF_{3SD}, g	-	0.032
**Validation Cohort**		
MF_{VA}	0.504	-
TF_{2SD}, g	-	0.039
GZF_{3SD}, g	-	0.050

Results of comparison of C statistics in relation to the primary endpoint are shown in terms of p values.

Table S5. Harrel C Statistics and Uno's C Statistics in the Derivation Cohort.

	Harrell's C statistic	Uno's C statistic
LVEF (%)	0.63	0.63
LVEF < 35%	0.58	0.58
MF_{VA}	0.72	0.68
MF_{VA} and LVEF < 35%	0.74	0.71
TF_{2SD}	0.75	0.70
TF_{2SD} and LVEF (%)	0.73	0.70
GZF_{3SD}	0.75	0.70
GZF_{3SD} and LVEF (%)	0.72	0.69

Results of Harrell's c statistics and Uno's c statistics for LVEF and myocardial fibrosis measures.

Table S6: Risk Reclassification Analyses: MF_{VA} over LVEF.

	**Model with LVEF<35 and MF_{VA}**			
**Model with LVEF<35%**	0-5%	10-15%	>15%	Total
0-5%	340	132		472
	2.65	12.9		5.51
	4.63	4.63		4.63
	2.1	10.8		4.53
5-10%	234	64	96	394
	2.14	15.6	11.5	6.60
	7.56	6.16	8.95	7.67
	2.93	12.9	16.3	7.8
Total	574	196	96	866
	2.44	13.8	11.5	6.00
	5.82	5.13	8.95	6.01
	2.44	11.46	16.3	6.02

	**NRI (95% CI)**
Event	0.462 (0.208, 0.682)
Non-event	0.376 (0.312, 0.440)
Overall	0.837 (0.580, 1.063)

Category-free net reclassification analyses of myocardial fibrosis by visual assessment (MF_{VA}) in relation to the primary composite arrhythmic endpoint in derivation sample, using LVEF<35% as the base model.

NRI = net reclassification improvement.

Table S7: Risk reclassification ana Risk Reclassification Analyses: TF_{2SD} mass over MF_{VA}.

	**Model with MF_{VA} and TF_{2SD} mass**			
**Model with MF_{VA}**	0-5%	5-10%	>15%	Total
0-5%	574			574
	2.44			2.44
	2.44			2.44
	2.44			2.44
10-15%		180	112	292
		9.44	18.8	13.0
		13.0	13.0	13.0
		9.45	18.8	13.0
Total	574	180	112	866
	2.44	9.44	18.8	6.00
	2.44	13.0	13.0	6.01
	2.44	9.45	18.8	6.01

	**NRI (95% CI)**
Event	0.077 (-0.252, 0.314)
Non-event	0.088 (0.021, 0.179)
Overall	0.165 (-0.219, 0.418)

Category-free net reclassification analyses of total fibrosis using the 2 SD method (TF_{2SD}) in relation to the primary composite arrhythmic endpoint in derivation sample, using myocardial fibrosis by visual assessment (MF_{VA}) as the base model.

NRI = net reclassification improvement.

Table S8: Risk Reclassification Analyses: GZF_{3SD} mass over MF_{VA}.

	**Model with MF_{VA} and GZF_{3SD} mass**			
**Model with MF_{VA}**	0-5%	5-10%	>15%	Total
0-5%	574			574
	2.44			2.44
	2.44			2.44
	2.44			2.44
10-15%		157	135	292
		8.28	18.5	13.0
		13.1	13.1	13.1
		8.29	18.6	13.1
Total	574	157	135	866
	2.44	8.28	18.5	6.00
	2.44	13.1	13.1	6.03
	2.44	8.29	18.6	6.02

	**NRI (95% CI)**
Event	0.231 (0.000, 0.460)
Non-event	0.042 (0.000, 0.135)
Overall	0.273 (0.054, 0.512)

Category-free net reclassification analyses of gray zone fibrosis using the 3 SD method (GZF_{3SD}) in relation to the primary composite arrhythmic endpoint in derivation sample, using myocardial fibrosis by visual assessment (MF_{VA}) as the base model.

NRI = net reclassification improvement.

Figure S1. Quantified TF and GZF in Relation to the Primary Endpoint.

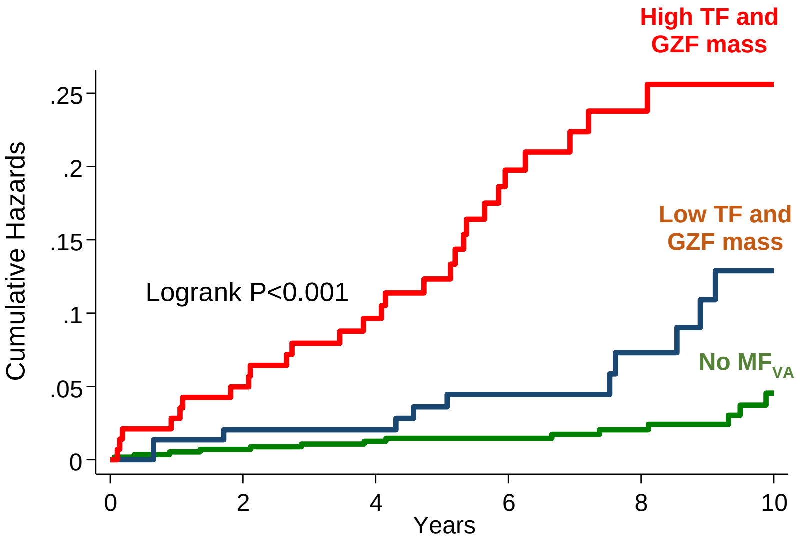

Number at risk

No MF$_{VA}$	574	553	505	405	269	111
Low TF and GZF mass	149	144	134	88	60	32
High TF and GZF mass	143	130	104	70	42	20

Cumulative hazard estimates of the primary arrhythmic endpoint in the derivation cohort, stratified according to the combination of total fibrosis (TF) mass using the 2 SD method (low: >0 and ≤10 g; high: >10 g) and gray zone fibrosis (GZF) mass using the 3 SD method (low: >0 and ≤3 g; high: >3 g). A ‘low TF and GZF’ means a TF >0 and ≤10 g and a GZF >0 and ≤3 g.

MF$_{VA}$ = myocardial fibrosis on visual assessment

Figure S2. Decision Curve Analysis.

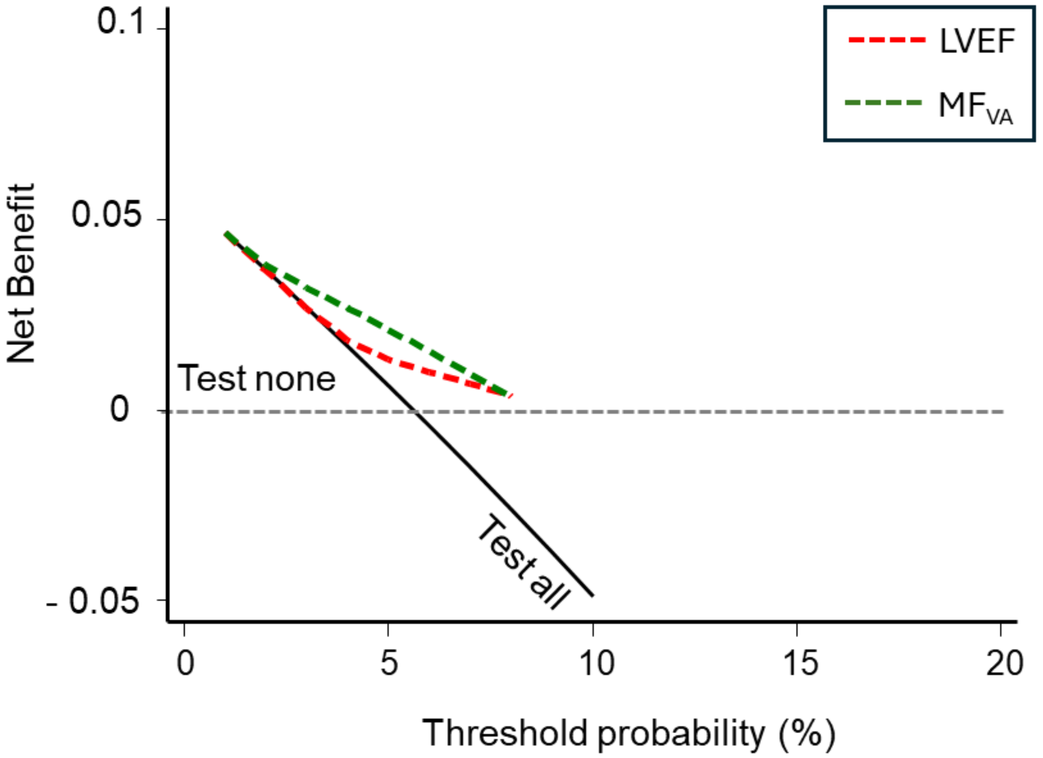

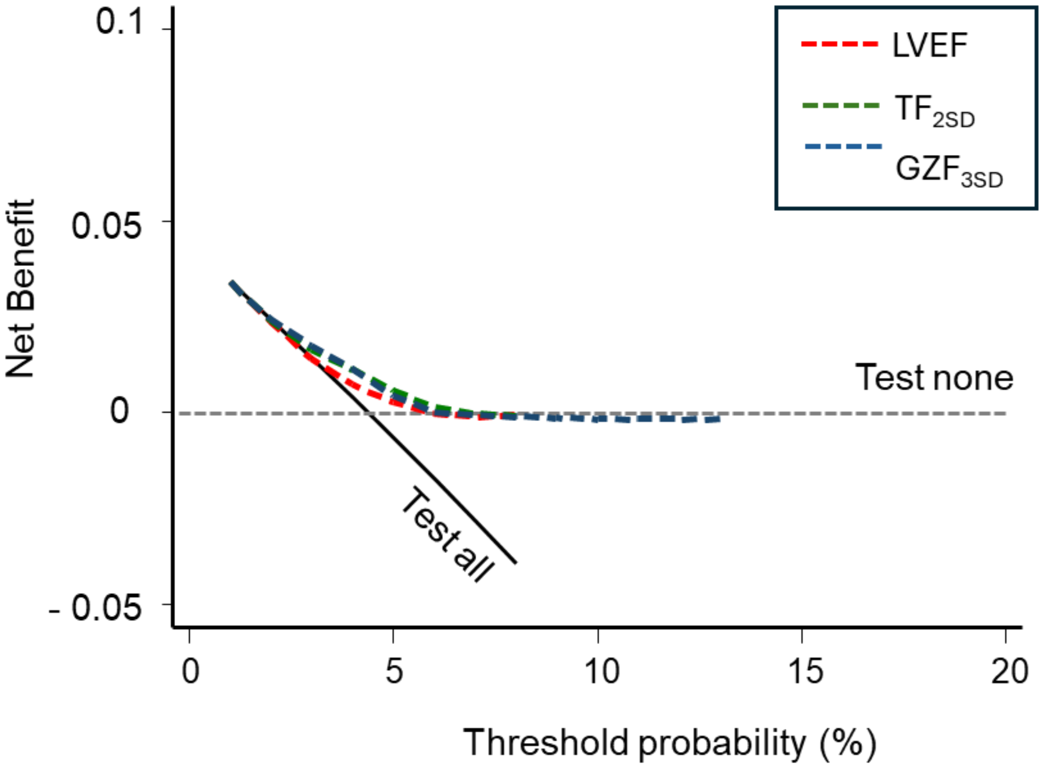

The graphs show decision curves in the validation cohort, comparing the net benefit of myocardial fibrosis (MF) (y axis) across different thresholds probabilities of the primary endpoint (x axis). The decision curve reflects the trade-off between true-positive predictions and false-positive predictions for a given strategy. The area under the decision curve quantifies the overall clinical utility of the predictive model. The dotted horizontal, gray line indicates the net benefit of not testing any patient ('test none') whereas the solid diagonal line shows the net benefit of testing all patients ('test all'). The dashed, coloured decision curves indicate the net benefit of using LVEF or MF measures in prediction models.

GZF_{3SD} = gray zone fibrosis according to the 3 SD method; MF_{VA} = myocardial fibrosis on visual assessment; SD = standard deviation; TF_{2SD} = total fibrosis according to the 2 SD method.
